# Ocean afforestation is a potentially effective way to remove carbon dioxide

**DOI:** 10.1038/s41467-023-39926-z

**Published:** 2023-07-20

**Authors:** Wei-Lei Wang, Mar Fernández-Méndez, Franziska Elmer, Guang Gao, Yangyang Zhao, Yuye Han, Jiandong Li, Fei Chai, Minhan Dai

**Affiliations:** 1 State Key Laboratory of Marine Environmental Science, College of Ocean and Earth Sciences, Xiamen University, Xiamen, 361102 China; 2 Alfred Wegener Institute Helmholtz Center for Polar and Marine Research, Bremerhaven, Germany; 3 School for Field Studies, Center for Marine Resource Studies, South Caicos, Turks and Caicos Islands; 4 State Key Laboratory of Numerical Modeling for Atmospheric Sciences and Geophysical Fluid Dynamics, Institute of Atmospheric Physics, Chinese Academy of Sciences, Beijing, China

**Keywords:** Marine biology, Carbon cycle

**arising from** L. T. Bach et al. *Nature Communications* 10.1038/s41467-021-22837-2 (2021)

Ocean afforestation has been proposed as a potential means of carbon dioxide (CO_2_) removal (CDR). However, a recent paper^1^ concludes that the efficacy of ocean afforestation may be reduced by 20–100% due to two biogeochemical feedbacks: calcification by epibionts and nutrient reallocation, it also suggests that atmospheric CO_2_ influx into the surface seawater, after CO_2_ fixation by *Sargassum*, takes 2.5–18 times longer than the time that surface seawater is in contact with the atmosphere. Here, we argue that these findings were technically biased due to utilization of selective data and evidence, and the conclusion drawn is thus insufficiently supported and maybe misleading. We support that thorough evaluations are essential before implementing any large-scale ocean-based CDR methods to avoid major negative consequences^2^, and ocean afforestation as a means of CDR is also such a case.

## Calcification

The calcification feedback argues that CO_2_ production due to the formation of carbonate shells attached to the surface of *Sargassum* can cancel most of the photosynthetic uptake of CO_2_. However, we point out that the calcification feedback is overestimated for the following four reasons.

Firstly, it is overestimated by including an outlier of the ratios of particulate inorganic carbon (PIC) to wet biomass weight. We ran a statistic test on the 13 ratios reported by Pestana^3^ using Matlab’s *isoutlier* function with the method of “median” and “quartiles”. Both methods identify the highest ratio of 21.4% as an outlier. Indeed, Pestana^3^ described the sample as “one exceptional sample” and excluded it in their analyses. The highest compensation effect of 57% is based on this outlier^1^. Given it being removed, the calcification compensation effect is decreased to 17.2% (6.6–34.8%). Furthermore, a recent study finds that calcareous epibiont content made up on average 2.09% (0.94–3.00%) of the wet weight of *Sargassum* collected from floating rafts near the eastern coastline of Quintana Roo, Mexico^4^. The new measurements conducted on *Sargassum* that travelled through the Great Atlantic *Sargassum* Belt (GASB), the same region studied by ref. ^1^, are therefore more relevant than the samples collected in another region^3^. If the new ratios were used, the compensation effect would be further reduced to 3.1% (1.3–4.5%).

Secondly, it is overestimated by ignoring the contribution of dissolved organic carbon (DOC) released by *Sargassum*. To test the calcification feedback, one should take into account all forms of organic carbon production including DOC, particulate organic carbon (POC), and small/suspended POC (sPOC), because the production of DOC and sPOC absorbs CO_2_ in exactly the same way as POC does. Bach et al.^1^ point out that DOC production exceeded POC buildup in the year 2018. However, they exclude its contribution by arguing that only a small fraction of DOC can survive remineralization for more than 20 years. This lifetime estimate is based on phytoplankton DOC^5^. A recent study^6^ suggests that a higher fraction (56–78%) of DOC released by *Sargassum horneri* is resistant to biological break-down. About 33% of DOC flux derived from macroalgae can be exported below the surface mixed layer and contribute to carbon sequestration^7^. If we take DOC export into account and use DOC:POC ratio (~0.33 × 1 Mt DOC [assuming a 33% export rate] compared to 0.81 Mt POC) during 2018 GASB, the calcification compensation effect will be further reduced to 0.9–3.2% (0.9–24.7% if Pestana data were included). Bach et al.^1^ did not consider contributions from DOC due to the challenges associated with monitoring this carbon pool. We disagree because being difficult to estimate does not mean that they can be disregarded.

Thirdly, it is overestimated by missing the avenue for carbon sequestration conducted by the export of sPOC, which are released during tissue fragmentation and breakage due to wave action and thallus decay. However, to our knowledge, sPOC production by *Sargassum* has yet to be determined. For cultivated kelp^8^, another brown seaweed, the sPOC can be 61% higher than harvested POC. If we assume that these two brown seaweeds share some similarities, sPOC production by *Sargassum* could be substantial. sPOC can be transported to the deep ocean via “particle injection pump”, and can be sequestered for decades to centuries^9^. Taking this into account, the calcification offset becomes negligible.

Furthermore, Bach et al.^1^ argue that the influx of atmospheric CO_2_ into surface water after CO_2_ fixation by *Sargassum* takes 2.5–18 times longer than CO_2_-deficient water remaining in contact with the atmosphere. However, this calculation was based on the behavior of phytoplankton that is distributed in the mixed layer and absorbs CO_2_ at depth given light is available. The mixed layer depth, the key parameter in the calculation, of 10–100 m is only applicable to phytoplankton and not to seaweeds that float at the top few meters. In any case, a coupled carbon cycle model should be used to conduct the calculation instead of the back-of-the-envelope calculation.

## Nutrient reallocation

The nutrient reallocation feedback mentions that if nutrients are not consumed by *Sargassum*, they would go to phytoplankton which is supposed to have lower PIC:POC ratios compared to *Sargassum*. However, it is clear that naturally occurring *Sargassum* blooms are not a suitable analogue for purposeful ocean afforestation, because one cannot expect any significant macroalgal growth in the open ocean due to micro- and macro-nutrient limitations, implying that the limiting nutrient iron in HNLC regions, or macronutrients N and P in subtropical gyres, will have to be provided artificially for ocean afforestation.

Ideally, in a fully operating aquafarm system nutrients can be recycled after harvesting and post-processing for biomethane production via anaerobic digestion^10^. The recycled nutrients will on one side alleviate nutrient limitation of *Sargassum*, on the other side will stimulate local primary production and enhance carbon export by phytoplankton. For CDR approaches it would be indeed desired that it is seaweed instead of phytoplankton that takes up the carbon and the nutrients due to its high carbon to nutrient ratio, larger size, faster sinking velocity, and more resistance to remineralization than phytoplankton.

Even if the nutrient reallocation effect is considered, the PIC compensation effect by phytoplankton is underestimated by picking a low PIC:POC ratio (0.01) for phytoplankton^11, 12^. Here their argument is that the nutrient reallocation decreases POC formation by phytoplankton as well as their PIC formation and thus CO_2_ production so that the “saved” amount of CO_2_ should be added to the theoretical CDR. However, values of PIC: POC are highly variable, and they range from 0.01 to 0.07 for the (sub)tropical Atlantic Ocean in their cited references. Other studies have detected an even higher basin mean ratio of 0.23–0.24^13^, ten times higher than that of *Sargassum* (0.02–0.07 from ref. ^4^). Since phytoplankton has similar or even higher ratios of PIC:POC, nutrient reallocation to *Sargassum* is favorable for CDR given the reasons stated above.

## Conclusions

Taken together, the conclusion of the paper that the two biogeochemical feedbacks reduce CDR efficacy of *Sargassum* by 20–100% is inaccurate and therefore misleading. However, we reiterate that research on ocean-based CDR is far from being enough and large-scale implementation needs careful evaluation. Ocean afforestation is one of the several potential ocean-based CDR approaches that need to be assessed and evaluated in terms of efficacy and environmental impact. And the evaluation should be carried out in a fair and balanced manner.

## Data Availability

Data sharing not applicable to this article as no datasets were generated during the current study.

## Code availability

Code for detecting outliers in a dataset is available in the following public GitHub repository: git@github.com:weileiw/IsOutlier.git.

## References

[1] Bach LT (2021). Testing the climate intervention potential of ocean afforestation using the Great Atlantic *Sargassum* Belt. Nat. Commun..

[2] Keller DP, Feng EY, Oschlies A (2014). Potential climate engineering effectiveness and side effects during a high carbon dioxide-emission scenario. Nat. Commun..

[3] Pestana H (1985). Carbonate sediment production by *Sargassum* epibionts. J. Sediment. Petrol..

[4] Salter MA, Rodríguez-Martínez RE, Álvarez-Filip L, Jordán-Dahlgren E, Perry CT (2020). Pelagic *Sargassum* as an emerging vector of high rate carbonate sediment import to tropical Atlantic coastlines. Glob. Planet. Change.

[5] Hansell DA (2013). Recalcitrant dissolved organic carbon fractions. Ann. Rev. Mar. Sci..

[6] Watanabe K (2020). Macroalgal metabolism and lateral carbon flows can create significant carbon sinks. Biogeosciences.

[7] Krause-Jensen D, Duarte CM (2016). Substantial role of macroalgae in marine carbon sequestration. Nat. Geosci..

[8] Zhang J (2012). Growth and loss of mariculture kelp *Saccharina japonica* in Sungo Bay. J. Appl. Phycol..

[9] Boyd PW, Claustre H, Levy M, Siegel DA, Weber T (2019). Multi-faceted particle pumps drive carbon sequestration in the ocean. Nature.

[10] N’Yeurt AdR, Chynoweth DP, Capron ME, Stewart JR, Hasan MA (2012). Negative carbon via Ocean Afforestation. Process Saf. Environ..

[11] Poulton AJ (2006). Phytoplankton mineralization in the tropical and subtropical Atlantic Ocean. Glob. Biogeochem. Cy.

[12] Sarmiento JL (2002). A new estimate of the CaCO_3_ to organic carbon export ratio. Glob. Biogeochem. Cy.

[13] Koeve W (2002). Upper ocean carbon fluxes in the Atlantic Ocean: The importance of the POC:PIC ratio. Glob. Biogeochem. Cy.

